# Photochemical reflectance index and its relation to photosynthetic characteristics under dynamic light environment

**DOI:** 10.1093/pcp/pcaf111

**Published:** 2025-09-11

**Authors:** Jing-Qi Zhang, Kouki Hikosaka, Hajime Tomimatsu

**Affiliations:** Graduate School of Life Sciences, Tohoku University, 6-3, Aoba, Aramaki, Aoba, Sendai 980-8578, Japan; Graduate School of Life Sciences, Tohoku University, 6-3, Aoba, Aramaki, Aoba, Sendai 980-8578, Japan; Graduate School of Life Sciences, Tohoku University, 6-3, Aoba, Aramaki, Aoba, Sendai 980-8578, Japan

**Keywords:** dynamic photosynthesis, nonphotochemical quenching, photochemical reflectance index, poplar, remote sensing, stomatal limitation

## Abstract

The photochemical reflectance index (PRI) is a normalized reflectance index that is expected to be useful for estimating photosynthetic activity based on remote-sensing images. Experimental and theoretical studies have examined how the PRI is related to photosynthesis, but they have been based on observations under steady-state light conditions. Photosynthetic systems display differential temporal responsiveness when exposed to variation in light intensity. Here, we examined the responses of the CO_2_ assimilation rate (*A*), quantum yield of PSII photochemistry (Ф_P_), nonphotochemical quenching (NPQ), and the PRI in two poplar species, one being a hybrid that does not close stomata in the dark (nonclosing type). When dark-adapted leaves were exposed to strong light (induction phase), the response time was Ф_P_ = NPQ < PRI < *A* for the normal type and Ф_P_ = NPQ < PRI = *A* for the nonclosing type. Consequently, the PRI–NPQ and the PRI–*A* relationships differed between the steady-state and induction phase. On the other hand, when the light-adapted leaves were transferred from dark to light, the time response was similar among Ф_P_, NPQ, and the PRI. Therefore, the PRI can be used to assess Ф_P_ and NPQ even under dynamic light conditions if light-adapted leaves are used. Our results imply that, following sudden increases in light intensity, CO_2_ assimilation in the normal type poplar is limited by stomatal conductance, and that PSII-related parameters, including the PRI, are temporally decoupled from *A*. Estimates of *A* based on the PRI would be overestimates under dynamic conditions, which needs to be taken into account when interpreting remote-sensing data.

## Introduction

Estimating vegetation productivity based on remote-sensing images, which offers advantages in terms of reduced labor requirements and broad-scale evaluation, is important for addressing global climate change, agricultural production, and ecosystem protection (Turner et al. 2003, Heinsch et al. 2006, Khanal et al. 2020). Vegetation indices such as the normalized difference vegetation index, which are based on the absorption spectra of photosynthetic pigment chlorophylls (Chl), have been widely used to assess leaf abundance in vegetation (Gao et al. 2000, Wang et al. 2004). However, such indices are insufficient to estimate vegetation carbon exchange because under unfavorable environmental conditions, photosynthetic activity often declines without a decrease in leaf number or Chl content (Gamon et al. 1995). Consequently, many researchers have sought to develop remote-sensing parameters that can detect photosynthetic activity directly.

The photochemical reflectance index (PRI), which measures the photosynthetic status of plants, is one such parameter. It is a normalized reflectance index based on reflectance at 531 nm, using reflectance at 570 nm as a reference (Gamon et al. 1993, Peñuelas et al. 1995). It indicates the de-epoxidation state of the xanthophyll cycle in PSII. In the dark, violaxanthin accumulates in leaves; under strong light, it is converted into zeaxanthin via antheraxanthin (de-epoxidation). When darkness returns, zeaxanthin is reversibly converted back to violaxanthin (epoxidation). Because absorbance at 531 nm differs between zeaxanthin and violaxanthin, the PRI can track the status of the xanthophyll cycle (Gamon et al. 1990, Ruban et al. 2001). Excess excitation energy is transferred from excited Chl to zeaxanthin and safely dissipated as heat (Lee et al. 2024). This process is a major component of heat dissipation and is known as nonphotochemical quenching (NPQ), which helps to protect PSII under stressful conditions in which excess light energy is present (Porcar-Castell et al. 2014, Bassi and Dall’Osto 2021). The PRI has been shown to correlate with the xanthophyll cycle status, quantum yield of CO_2_ assimilation, quantum yield of PSII photochemistry (Ф_P_), and NPQ (Gamon et al. 1990, 1992, 1997, Stylinski et al. 2002, Evain et al. 2004, Sarlikioti et al. 2010, Garbulsky et al. 2011, Kováč et al. 2018, Sancho-Knapik et al. 2018, Sukhova and Sukhov 2018, Hikosaka and Noda 2019, Kohzuma et al. 2021). Therefore, it is considered an effective metric for assessing photosynthetic activity and plant stress via remote-sensing images.

Previous modeling studies have used relationships between photosynthetic parameters and the PRI that were obtained under steady-state conditions (e.g. Atherton et al. 2016, Hikosaka and Noda 2019). However, light conditions in the field are not always stable and often change dynamically due to factors such as moving clouds, wind, or shading by other objects (e.g. Tang et al. 1988). Although photosynthesis is light-dependent, the CO_2_ assimilation rate does not respond instantly to changes in light (Pearcy 1990, Way and Pearcy 2012, Kaiser et al. 2015, Tomimatsu and Tang 2016). In darkness, some photosynthetic components such as ATPase, Rubisco, and certain Calvin-Benson cycle enzymes become inactivated, and stomata close (Matthews et al. 2020, Gurrieri et al. 2021). When dark-adapted leaves are exposed to strong light, CO_2_ assimilation gradually increases (induction phase). In the early fast phase of induction, thylakoid reactions—including PSII photochemistry—are quickly activated, but Calvin-Benson cycle activation is slower and limits the assimilation rate (Pearcy 1990). Stomata begin to open after the light increases, but their rate of change lags behind the activation of photosynthetic enzymes (Kirschbaum et al. 1988, Shimazaki et al. 2007, McAusland et al. 2016). In the later slow phase, CO_2_ assimilation becomes limited by CO_2_ diffusion (Kirschbaum et al. 1988, Pearcy 1990). During this process, thylakoid electron transport, Calvin-Benson cycle activity, and CO_2_ diffusion are decoupled. Conversely, when light-adapted leaves transition from shade to sun, the increase in CO_2_ assimilation is faster than that in dark-adapted leaves, as most reactions have already been activated (Rijkers et al. 2000). When the light intensity drops suddenly, CO_2_ assimilation also declines rapidly, but not instantaneously. Post-illumination CO_2_ assimilation or a CO_2_ burst can occur, depending on the accumulation of photosynthetic and photorespiratory intermediates (Pearcy 1990).

Temporal changes in energy partitioning in PSII under dynamic light conditions are also complex. In healthy leaves of terrestrial plants, NPQ is achieved not only via zeaxanthin but also through PsbS, a subunit of PSII (Bassi and Dall’Osto 2021). Studies using mutants that lack de-epoxidation activity (*npq1*; zeaxanthin not formed) or PsbS (*npq4*) have shown that under steady-state conditions, both contribute comparably to NPQ (Crouchman et al. 2006, Dall’Osto et al. 2017). However, NPQ induced via PsbS is activated more rapidly than that driven by zeaxanthin (Nilkens et al. 2010, Lee et al. 2024), implying that zeaxanthin contributes less during the induction phase. Kohzuma and Hikosaka (2018) showed that the PRI responded to light intensity in the wild type and *npq4*, but not in *npq1*, indicating that the PRI reflects xanthophyll de-epoxidation more than PsbS activity. Taken together, these findings imply that PRI induction is slower than NPQ due to delayed xanthophyll cycle activation.

Models for estimating photosynthetic rates via remote sensing have generally been built on steady-state measurements (van der Tol et al. 2009, 2014, Atherton et al. 2016, Hikosaka and Noda 2019). Under such conditions, reaction rates across photosynthetic components are governed by feedback mechanisms. For instance, under low CO_2_ concentrations, CO_2_ assimilation is limited by the RuBP carboxylation rate, while other processes maintain higher potential (Farquhar et al. 1980). In this situation, processes such as PSII photochemistry are downregulated to coordinate RuBP regeneration with Rubisco activity. This coordination allows estimation of CO_2_ assimilation rates using PSII activity or the electron transport rate (Genty et al. 1989). However, a study on the different responses of photosynthetic processes under fluctuating light indicated that component activities may become uncoupled (Pearcy 1990), and models based on steady-state conditions may be inaccurate in dynamic light environments.

In the present study, we investigated temporal changes in the CO_2_ assimilation rate, Ф_P_ (the quantum yield of photochemistry, as the ratio of the number of photochemical events to the number of photons absorbed by the PSII), NPQ, and the PRI under dynamic light conditions: when dark-adapted leaves are exposed to strong light (induction phase), when leaves are transferred from strong light to dark (deactivation phase), and when light-adapted leaves are transferred from dark to strong light (recovery phase). We used two poplar species: one with the normal stomatal response to light change (I-214; normal type), and the other a hybrid that does not close stomata in the dark and maintains high stomatal conductance under dark conditions (Peace; nonclosing type) (Tomimatsu and Tang 2012). This comparison allowed us to assess how stomatal limitation feedback influenced other photosynthetic processes. Based on findings from previous studies, we address the following hypotheses: under dynamic light conditions, feedback from stomatal limitation does not occur, and the response time of CO_2_ assimilation is very different from that of PSII activity and the PRI; under dynamic light conditions, the response time of the PRI is longer than that of NPQ; and as a result of different response times, the relationships between the PRI and photosynthetic parameters differ between dynamic and steady-state conditions. We discuss the usefulness of the PRI for estimating photosynthetic characteristics under fluctuating light.

## Results and Discussion

**Figure 1:**
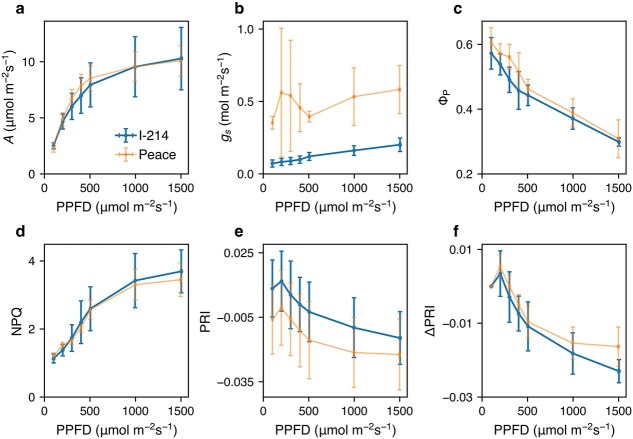
Light response of the characteristics under the steady state conditions in I-214 and Peace. *A*, CO_2_ assimilation rate (a); *g*_s_, stomatal conductance (b); Φ_P_, quantum yield of PSII photochemistry (c); NPQ, nonphotochemical quenching (d); PRI, photochemical reflectance index (e); ΔPRI, corrected PRI (f). The bar denotes SD. The sample size was 7 and 6 for I-214 and Peace, respectively.

Under the steady state, the light-response curve of the CO_2_ assimilation rate (*A*) was similar between I-214 (normal type) and Peace (nonclosing type) poplar, whereas stomatal conductance (*g*_s_) was much higher in the latter (Fig. 1). Φ_P_ and NPQ decreased and increased with increasing light, respectively, and their light responses were also similar in the two poplar types. PRI values decreased with increasing light and were higher in I-214. However, the corrected PRI (ΔPRI, PRI minus initial PRI) showed similar light dependence between the two types. The intercept of the relationship between NPQ and the PRI differs among leaves depending on pigment composition, but the slope is generally consistent (Rahimzadeh-Bajgiran et al. 2012, Hmimina et al. 2015, Tsujimoto and Hikosaka 2021, Nakamura et al. 2024). Our results align with those findings.

**Figure 2:**
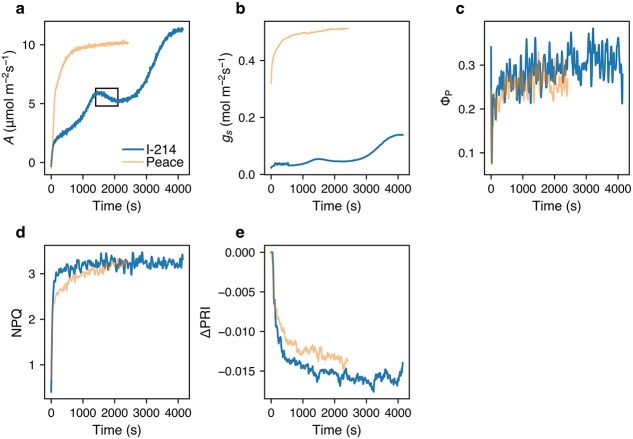
Example of the changes in characteristics in dark-adapted leaves of I-214 and peace in the induction phase. *A*, CO_2_ assimilation rate (a); *g*_s_, stomatal conductance (b); Φ_P_, quantum yield of PSII photochemistry (c); NPQ, nonphotochemical quenching (d); ΔPRI, corrected PRI (e). Representative results from one of the studied leaves are shown. The box in (a) is the window in which *A* decreased with time during the induction (oscillation). This window was used to assess whether oscillation in *A* is coupled with the PSII activity.

In the induction phase (dark-adapted leaves transferred from dark to light), *A* increased over time, with a much faster increment in Peace than in I-214 (Fig. 2). Some leaves exhibited oscillations during the increase in *A*. The faster increase in *A* in Peace was attributed to greater *g*_s_, even in its dark-adapted leaves. Φ_P_ was high in darkness, decreased rapidly upon illumination to a minimum, and then gradually increased. NPQ was low at light onset, followed by a rapid increase. No significant difference in NPQ response times were observed between I-214 and Peace. ΔPRI values decreased rapidly, and the response times of the two poplars were similar.

**Figure 3:**
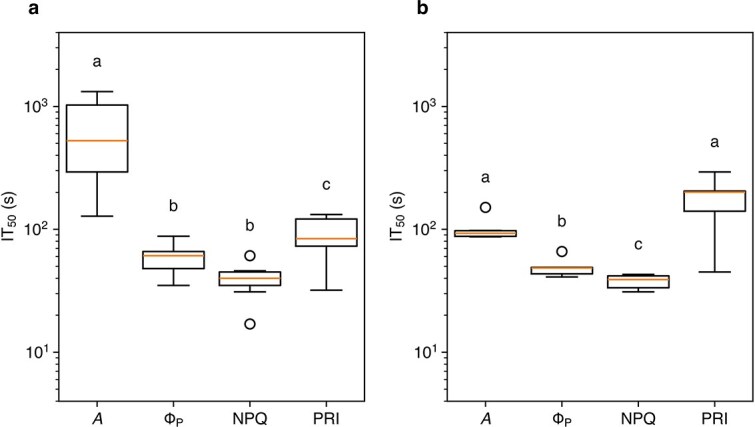
Box plot of the response time (IT_50_) in the induction phase in I-214 (a) and Peace (b). *A*, CO_2_ assimilation rate; Φ_P_, quantum yield of PSII photochemistry; NPQ, nonphotochemical quenching; PRI, photochemical reflectance index. Different alphabets above the box denote significant differences tested by the paired *t*-test with post hoc test according to Holm (1979).

We calculated IT_50_, the time required for a 50% change in a parameter from the start of induction (see *Materials and Methods* for details). For Φ_P_, the lowest value was taken as the initial value. In I-214, IT_50_ was much longer for *A* than for Φ_P_, NPQ, and ΔPRI. The IT_50_ of ΔPRI was significantly longer than that of Φ_P_ and NPQ (Fig. 3). In Peace, that of *A* was much shorter than that in I-214 and was comparable to that of ΔPRI. The IT_50_ of ΔPRI was significantly longer than that of Φ_P_ and NPQ.

**Figure 4:**
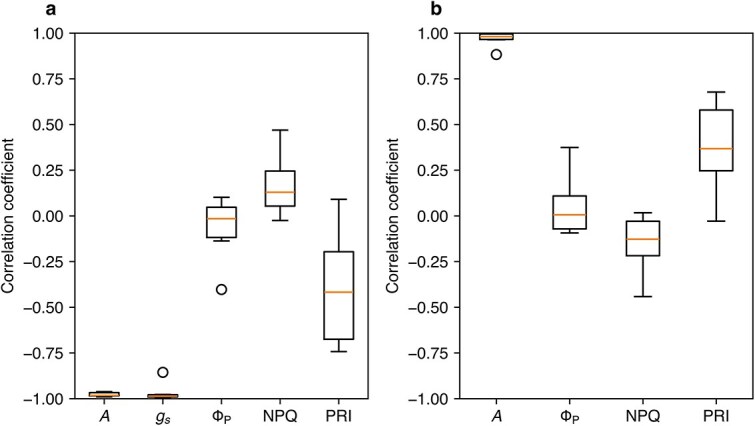
Box plot of Pearson’s correlation coefficient for the relationship between the photosynthetic variable and time (a); or between the photosynthetic variable and stomatal conductance (b), when the CO_2_ assimilation rate (a) decreased with time in the induction phase (oscillation) in I-214 (see the box in Fig. 2a). Φ_P_, quantum yield of PSII photochemistry; NPQ, nonphotochemical quenching; PRI, photochemical reflectance index.

Next, we examined relationships among photosynthetic characteristics during stomatal oscillations. Specific time windows in which *A* decreased over time were selected for some leaves (Fig. 2a). During this window, *A* and *g_s_* showed a negative correlation with time (Fig. 4). The correlation between Φ_P_ and time or *g_s_* was not significant (Fig. 4), as Φ_P_ was already stable (Fig. 2), and this was also true for NPQ (Fig. 4). These findings imply that PSII light partitioning was decoupled from *A* during oscillation. The PRI exhibited no or a negative correlation with time in this window, indicating that the PRI was also decoupled from *A* during oscillation.

Our results imply that, in the induction phase, the response time of the PRI was slightly longer than those of PSII activities (Φ_P_ and NPQ) in both I-214 and Peace (Fig. 3), as hypothesized. This may be partially explained by differences in response times between PsbS-dependent and zeaxanthin-dependent NPQ. Using *Arabidopsis* mutants, Nilkens et al. (2010) analyzed temporal changes in NPQ after illumination. They calculated the response time (*τ*) using an exponential function [1 − exp(−*t/τ*)], where *t* is the time after illumination. Applying this function to our results, IT_50_ = 0.69*τ*. In Nilkens et al. (2010), the *τ* of NPQ in the wild type was 59 s, corresponding to 40.7 s of IT_50_, which is similar to our value (39.7 s in I-214). The *τ* values of NPQ in mutants lacking de-epoxidase activity (*npq1*) and lacking PsbS (*npq4*) were 9 s and 460 s, respectively, equivalent to IT_50_ values of 6 s and 317 s, indicating that zeaxanthin conversion responds much more slowly than PsbS protonation. Because the PRI is considered to primarily reflect xanthophyll de-epoxidation status, this is consistent with the longer PRI response time compared to NPQ. However, the average IT_50_ of the PRI in our study was 131 s, much shorter than that of NPQ in *npq4*, implying that the PRI response time cannot be explained solely by xanthophyll dynamics. Zolin et al. (2024) suggested that rapid changes in the PRI may also involve PsbS, as the latter promotes aggregation of LHCII, altering absorbance at 535 nm (Li et al. 2000, Duffy et al. 2010, Johnson and Ruban 2010, Johnson and Ruban 2014). Thus, early changes in the PRI may be partially attributable to PsbS. However, this interpretation conflicts with the observation that the PRI in *npq1* under steady-state conditions is largely independent of light intensity (Kohzuma and Hikosaka 2018). Additionally, most knowledge of NPQ dynamics is based on *Arabidopsis*, and induction rates may differ among species. Further studies are needed to understand better how PsbS and xanthophylls influence the PRI under dynamic light conditions.

Slower induction of *A* in I-214 than in Peace implies that, in the induction phase, *A* in I-214 is strongly limited by *g*_s_ (Figs. 2 and 3). In I-214, the IT_50_ of *A* was much longer than that of PSII parameters and the PRI. Furthermore, PSII parameters and PRI values were not related to the oscillation in *A* and *g*_s_ as discussed above (Fig. 4). These results imply that thylakoid reactions such as PSII activity and electron transport are decoupled from CO_2_ assimilation in the induction phase. In Peace, induction of *A* was slightly slower than Φ_P_ and NPQ, even though it was not limited by *g*_s_. This may have been due to limitations in downstream photosynthetic reactions, such as Calvin-Benson cycle enzymes. The *A*–*C*_i_ curve of Peace in a previous study (Tomimatsu et al. 2019) implies that at ambient CO_2_ concentration *A* is limited by Rubisco activity. The induction of *A* in Peace in this study (IT_50_ = 101.5 s) was faster than Rubisco activation in *Alocasia macrorrhiza* (IT_50_ = 2.5 min; Seemann et al. 1988). This indicates that Peace has a higher Rubisco activation rate compared to *A. macrorrhiza*, implying interspecific variation in Rubisco activation.

**Figure 5:**
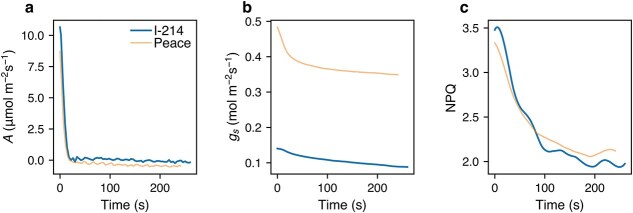
Example of the changes in characteristics in leaves of I-214 and Peace in the deactivation phase. *A*, CO_2_ assimilation rate (a); *g*_s_, stomatal conductance (b); NPQ, nonphotochemical quenching (c); representative results from one of the studied leaves are shown.

**Figure 6:**
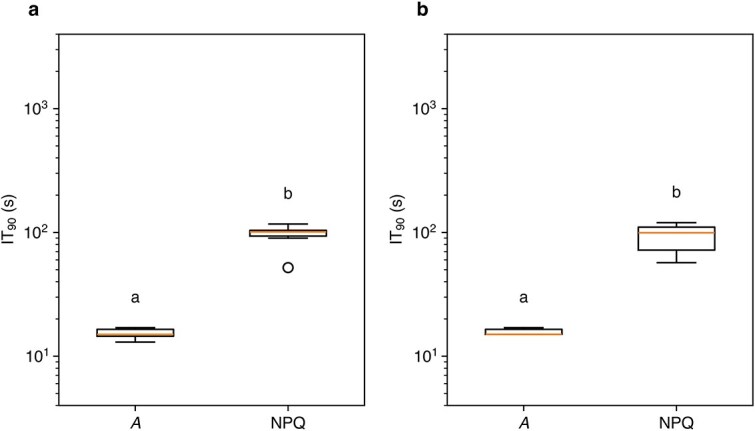
Box plot of the response time (IT_90_) in the deactivation phase in I-214 (a) and Peace (b). *A*, CO_2_ assimilation rate and NPQ, nonphotochemical quenching. Different alphabets above the box denote significant differences tested by the paired *t*-test.

In the deactivation phase (light-adapted leaves transferred from strong light to dark; Supplementary Fig. S2), we analyzed the dynamics of gas exchange (*A* and *g*_s_) and NPQ only, because PRI values were not measured and *F_s_* values were unreliable. *A* declined very quickly, whereas *g*_s_ and NPQ declined slowly (Fig. 5). IT_90_, the time to reach 90% of the steady-state value, was longer for NPQ than for *A* in both I-214 and Peace (Fig. 6). The rapid decline in *A* is attributable to the termination of energy input (light), independent of *g*_s_ and NPQ. Stomatal closure proceeds slowly due to multiple regulatory steps (Shimazaki et al. 2007). The decline in NPQ is also slow, mainly due to low zeaxanthin epoxidase activity (Demmig-Adams 1990), resulting in a substantial loss of photochemical energy (Werner et al. 2001, Zhu et al. 2004).

**Figure 7:**
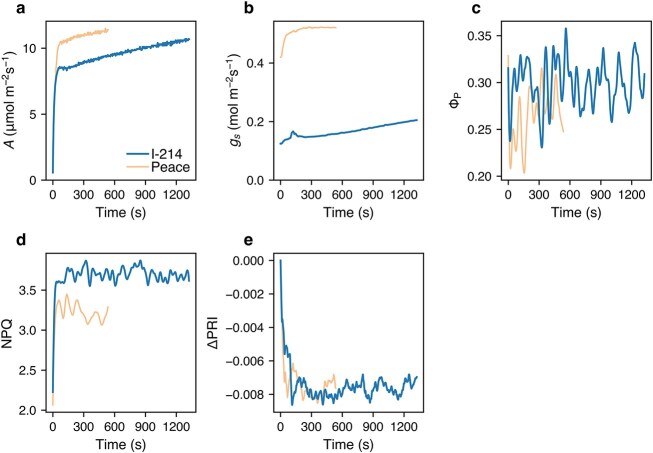
Example of the changes in characteristics in light-adapted leaves of I-214 and Peace in the recovery phase. *A*, CO_2_ assimilation rate (a); *g*_s_, stomatal conductance (b); Φ_P_, quantum yield of PSII photochemistry (c); NPQ, nonphotochemical quenching (d); ΔPRI, corrected PRI (e). Representative results from one of the studied leaves are shown.

**Figure 8:**
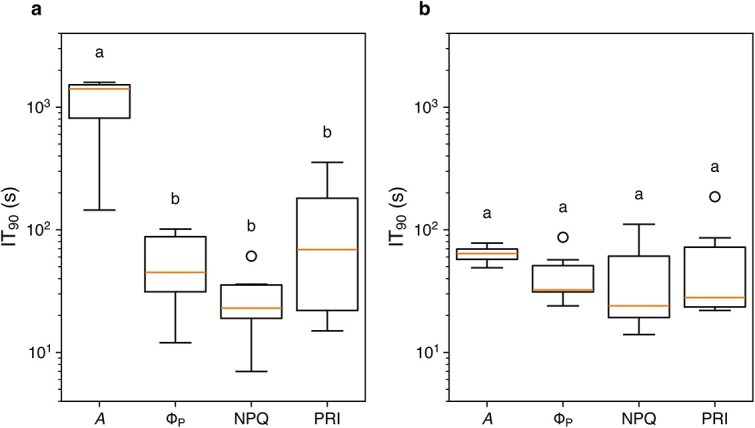
Box plot of the response time (IT_90_) in the recovery phase in I-214 (a) and Peace (b). *A*, CO_2_ assimilation rate; Φ_P_, quantum yield of PSII photochemistry; NPQ, nonphotochemical quenching; PRI, photochemical reflectance index. Different alphabets above the box denote significant differences tested by the paired *t*-test with post hoc test according to Holm (1979).

In the recovery phase (light-adapted leaves transferred from dark to light), all parameters responded more quickly than in the induction phase (Fig. 7). In I-214, the IT_90_ of *A* was slightly longer than that of other parameters, which had similar IT_90_ values (Fig. 8). In Peace, the IT_90_ was similar across all parameters, likely because they were already activated at the onset of the recovery phase. Sassenrath-Cole and Pearcy (1994) reported that Rubisco deactivation in *Glycine max* is very slow, with an IT_50_ of about 24 min. Thus, we infer that our poplars maintained high Rubisco activity even after 4 min of darkness, allowing carbon assimilation to resume immediately upon reillumination. These findings imply that, in this phase, parameters other than stomatal conductance exhibited similar response times, and the CO_2_ assimilation rate of normal plants was limited solely by stomatal conductance.

**Figure 9:**
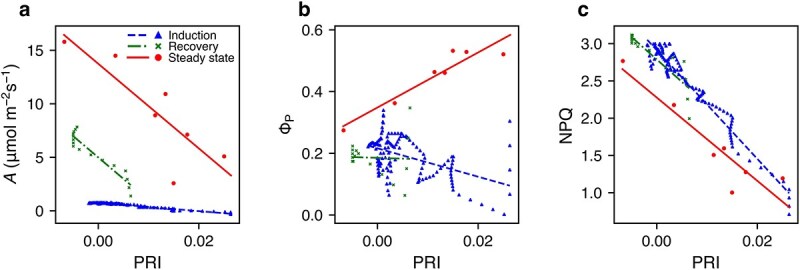
Examples of the relationships between *A* (CO_2_ assimilation rate) and PRI (photochemical index) (a), Φ_P_ (quantum yield of PSII photochemistry) and PRI (b), NPQ and PRI (c) in different phases in I-214. Blue, green, and red symbols represent induction, recovery, and steady state phases, respectively. Lines are linear regression. Representative results from one of the studied leaves are shown. The overall statistical analysis is shown in Table 1. (a) Induction: *A* = −39.1PRI + 0.75, *r*^2^ = 0.94, *P* < 0.001, recovery: *A* = −399PRI + 4.99, *r*^2^ = 0.89, *P* < 0.001, steady-state: *A* = −396PRI + 13.7, *r*^2^ = 0.712, *P* < 0.05 (b) induction: Φ_P_ = −4.6PRI + 0.216, *r*^2^ = 0.229, *P* < 0.001, recovery: Φ_P_ = −0.506PRI + 0.185, *r*^2^ = 0.001, *P* = 0.889, steady-state: Φ_P_ = 8.91PRI + 0.384, *r*^2^ = 0.889, *P* < 0.01 (c) induction: NPQ = −73PRI + 2.91, *r*^2^ = 0.95, *P* < 0.001, recovery: NPQ = −60.0PRI + 2.79, *r*^2^ = 0.769, *P* < 0.001, steady-state: NPQ = −55.9PRI + 2.28, *r*^2^ = 0.856, *P* < 0.01.

As a result of this variation in response time, the relationships among photosynthetic components differed between the induction, recovery, and steady-state phases. Figure 9 shows an example of an I-214 individual, and Table 1 shows the statistical results of the linear mixed model (LMM) to all I-214 (see *Materials and Methods*). The *A*–PRI relationship was negative in all phases, with a gentler slope in induction compared to the steady-state, because PRI values declined more rapidly while *A* remained low in the early stages. The Φ_P_–PRI relationship was positive under steady-state conditions, reflecting higher Φ_P_ under low light, whereas it was negative in the induction and recovery phases, due to increasing Φ_P_ under constant light. The NPQ–PRI relationship was consistently negative, as demonstrated in previous studies. Due to the slower PRI response in the induction phase, the NPQ–PRI slope differed from that of the steady-state phase. By contrast, the NPQ–PRI relationship did not differ in either slope or intercept between the recovery and steady-state phases because the PRI and NPQ exhibited similar response times during these periods.

**Table 1:** Coefficient of the LMM used to assess differences in the regression line between phases in I-214. A, Φ_P_, and NPQ are the CO_2_ assimilation rate, quantum yield of PSII photochemistry, and NPQ, respectively. The first row of the table is the overall intercept of the model. The second is the coefficient of the photochemical reflectance index (PRI), which indicates the degree of influence of the index on the intercept of the model. The third is the coefficient of the induction phase, which indicates the influence of the induction phase on the intercept of the model compared to the steady state. The fourth row is similar, indicating the influence of the recovery phase on the intercept of the model. The fifth row shows the mixed effect of the PRI and the induction phase, which indicates the influence of the induction phase on the slope of PRI–NPQ values. The sixth row shows the influence of the recovery phase on the slope of PRI–NPQ values.

Dependent variable	*A*	Φ_P_	NPQ
Intercept	7.10^***^	0.437^***^	1.97^**^
PRI	−137.7^***^	−1.200 NS	−107.5^***^
Induction	−5.72^***^	−0.293^***^	0.007 NS
Recovery	−3.25^***^	−0.277^***^	−0.0162 NS
PRI: induction	−63.6^***^	−6.50^***^	6.08^*^
PRI: recovery	−35.8^**^	−6.16^***^	−0.384 NS

NS *P* > 0.05.

^^*^^ *P* < 0.05.

^^**^^ *P* < 0.01.

^^***^^ *P* < 0.001.

The mechanistic model for predicting *A* from the PRI involves several steps (Hikosaka and Tsujimoto 2021). First, light partitioning within PSII is estimated under the assumption that NPQ is negatively related to the PRI. Lower PRI values indicate greater energy allocation to heat dissipation, resulting in lower Φ_P_ values (van der Tol et al. 2014). The thylakoid electron transport rate can be estimated from Φ_P_ and the light absorbed by PSII (Schreiber et al. 1995). Then, if the intercellular CO_2_ concentration is known, *A* can be calculated using the biochemical model of photosynthesis (Farquhar et al. 1980). These relationships hold under steady-state conditions, due to coordination among photosynthetic components. When one process is environmentally limited, other processes are typically downregulated in response. However, our findings imply that such coordination may break down under dynamic light conditions.

What happens when *A* is estimated from the PRI under dynamic light using models derived from steady-state observations? Under low steady-state light, high PRI values correspond to low heat dissipation and low *A*. But when light suddenly increases, the PRI decreases faster than *A* increases, resulting in overestimation of *A*. Although we did not consider solar-induced Chl fluorescence, another method for estimating photosynthetic activity in remote sensing, a similar logic applies, because its modeling approach also depends on coupled physiological responses (Hikosaka and Tsujimoto 2021).

To address this issue, it is important to consider physiological decoupling when interpreting remote-sensing data collected under field conditions. Ideally, observations should be made under stable sky conditions. Developing new models that account for dynamic responses is feasible but would require detailed information about PSII function, light-activated enzymes, and stomatal behavior. On the other hand, our results show that response time differences were smaller in the recovery phase than in induction. In typical remote-sensing scenarios, leaves are light-adapted and not in the dark. While *A* might still be overestimated, the effect is expected to be relatively small. Moreover, the PRI can be used to estimate NPQ under dynamic conditions, provided that measurements are taken from light-adapted leaves.

This study qualitatively pointed out the decoupling of PRI and NPQ during the induction phase and attributed it to the difference between PsbS and the xanthophyll cycle. However, we were unable to quantitatively indicate why the IT_50_ of PRI in *Populus* (131 s) was so different from that of PsbS (6 s) and xanthophyll cycle (317 s) in *Arabidopsis*. This may be due to interspecific differences or the effect of PsbS on PRI. More detailed physiological studies are needed in the future.

## Conclusion

Our study demonstrated that response times to dynamic light differ among photosynthetic characteristics, including the PRI. After a sudden increase in light, the PRI responded more slowly than PSII photochemistry and NPQ, likely because the former is primarily regulated by zeaxanthin-dependent NPQ, induction of which lags behind that of PsbS-dependent NPQ. In the normal type poplar (I-214), the response of *A* to increased light was substantially slower than that of the PRI, resulting in overestimation of the former when predicted from the PRI using models based on steady-state relationships. However, response time differences among photosynthetic characteristics were smaller in light-adapted leaves than in dark-adapted ones. Because remote sensing typically targets light-adapted leaves, errors in estimating photosynthetic status under dynamic conditions may be minor, even when steady-state assumptions are applied.

## Materials and Methods

### Plants and growth conditions

We used two poplar species, *Populus euramericana* cv. I-214 (herein, I-214) and an ABA-unresponsive hybrid (*Populus koreana × trichocarpa* cv. Peace; herein, Peace), with different stomatal behaviors to explore changes in PSII parameters and the PRI under varying stomatal conditions. The stomata in I-214 open in light and close in darkness, as in many plant species, whereas those in Peace remain open even in the dark (Furukawa et al. 1983, 1990, Ridolfi et al. 1996). Poplar branches were provided by the National Institute for Environmental Studies (Japan), and cuttings were sampled from them in April 2024. Branches were cut into strips approximately 30 cm in length, and growth promoter (Luton, Sumitomo Chemical) was applied to the cut surfaces. Then each cutting was placed into a 4-L pot (one individual per pot) filled with soil (vermiculite and Nippi soil mixed at a 1:1 ratio). All poplars were grown in a greenhouse located in Sendai, Japan (38°15′15″N, 140°50′50″E, 139 m above sea level) under natural sunlight, without regulation of temperature or CO_2_ concentration. Watering was performed once every 2 weeks before leafing out, once a week during initial leafing, and three times per week once the plants reached ~70 cm height and had developed lush branches and leaves.

Measurements were conducted in late September and early October 2024. The day before measurement, the plants were moved with their pots from the greenhouse into a growth chamber. They were placed in a closed, completely dark environment overnight and watered adequately to induce dark adaptation. Ambient temperature and CO_2_ concentration in the chamber were maintained at 25°C and 420 ppm, respectively. For measurement, the youngest fully expanded and uninjured leaf was selected from each individual. The target leaf was washed with water, dried with a towel, and then fully wrapped in aluminum foil until just before measurement to maintain dark adaptation.

### Experimental equipment and setup

Gas exchange rates were measured using a portable photosynthesis system (Li-6400, Li-Cor Biosciences, USA). Chl fluorescence was measured using a MINI-PAM-II chlorophyll fluorescence device (Walz, Effeltrich, Germany). The reflectance spectrum was measured using a high-resolution spectrometer (Ocean HR, Ocean Optics, USA). Measurements with these three instruments were performed using an improved version of a previously developed system that enables simultaneous measurements (Tsujimoto and Hikosaka 2021). In the improved setup, the two optical fibers used to detect Chl fluorescence and spectral reflectance are positioned closer together than before, and their angles are adjusted. Lighting for the measurements was provided by an LED ceiling light (Nippon Medical & Chemical Instruments Co., Ltd., PFQ-600DT), with light intensity controlled by a power unit (Nippon Medical & Chemical Instruments Co., Ltd., PFQ-1200BB). In the following text, “fluorometer” refers to the MINI-PAM-II, and “spectrometer” refers to the Ocean HR. During the experiment, each poplar leaf was clamped into a 2 cm × 3 cm leaf chamber of the Li-6400. The upper portion of the chamber was covered with an acrylic plate, into which two optical fibers connected to the spectrometer and fluorometer were inserted. This configuration allowed direct measurement of reflected light and fluorescence from the leaf surface, without interference from the acrylic plate (Supplementary Fig. S1).

### Measurement

This experiment used LED lighting. Because the spectral shape of LED light may vary across light intensities, we measured LED spectra at different intensities to calculate leaf reflectivity accordingly. The spectrum of incident light was initially represented by the reflectance spectrum of a white board. However, under strong light, the reflectance of the whiteboard exceeded the upper limit of the spectrometer. Therefore, we adopted an alternative solution. A white or gray board was clamped into the Li-6400 leaf chamber, positioned identically to the later placement of poplar leaves. The LED power was adjusted until the Li-6400 light sensor read 200 μmol m^−2^ s^−1^, allowing measurement of the white or gray board reflectance spectrum under weak light conditions. Then we calculated R_gray,white_, the ratio of the reflectance of the gray board relative to the white board. Because R_gray,white_ depended only on the physical properties of the boards, it did not vary with light intensity. Subsequently, we changed the light intensity (1500, 1000, 500, 400, 300, 200, 100 μmol m^−2^ s^−1^), measured the gray board spectra under each light level, and divided the values by R_gray,white_ to derive the corresponding white board spectra. These whiteboard spectra were used to represent the incident light spectra. The spectral shape is shown in Supplementary Fig. S3.

After measuring the incident spectra, we removed the gray board from the leaf chamber and unwrapped the aluminum foil from the poplar leaf, still under dark conditions. The leaf was placed into the chamber without exposure to light. Measurement conditions were 420 ppm CO_2_, 50%–70% RH in the chamber, 25°C leaf temperature, and 500 μmol s^−1^ flow rate. Measurements were performed using the illumination pattern shown in Supplementary Fig. S2. We tracked changes in gas exchange rates, Chl fluorescence, and reflectance spectra under the following conditions: in dark-adapted leaves exposed to strong light (induction phase), in light-adapted leaves exposed to darkness (deactivation phase), and in light-adapted leaves re-exposed to strong light (recovery phase). We also determined these parameters at different light intensities under steady-state conditions (steady-state phase).

In the induction phase, measurement durations varied among individuals because the time to reach steady state was not uniform. Measurement in the induction phase was stopped when *A* reached steady state, defined as no increase exceeding 3% within a 5-min interval. The deactivation phase was fixed at 4 min, because the deactivation process is rapid, and this duration was sufficient for all leaves to reach steady state. Recovery phase measurements followed the same principle as the induction phase. In the steady-state phase, each light intensity was applied for 5 min, and the average of the final 30 s in each step was used as the steady-state data.

During measurements, the spectrometer and fluorometer ran continuously under automated control. Measurement intervals were shorter under high light intensity and longer under low light. This design reflects the requirement for longer exposure times at low light intensity to ensure sufficient photon detection for accurate readings. The corresponding relationship between PPFD and exposure time is shown in Supplementary Table S1.

Reflectance values were calculated as the average of three measurements. After the spectrometer measurement was completed, the fluorometer emitted a pulse measuring light to excite Chl and then a saturating flash to initiate the photochemical reaction. Following Chl fluorescence measurement, the reflectance spectrum measurement was repeated. The automation program, written in C language, was configured to detect fluorometer activity and ensure spectrum acquisition only after modulated light was fully extinguished, thus avoiding contamination from the measuring light.

Gas exchange rates were recorded at 4-s intervals. Because the Li-6400 measurement interval did not match that of the spectrometer and fluorometer, linear interpolation at 1-s intervals was used to align paired datasets.

### Parameters

The quantum yield of PSII photochemistry (Φ_P_) was obtained according to Genty et al. (1989):


(1) \begin{equation*} {\varPhi}_P=\frac{F_m^{\prime }-{F}_s}{F_m^{\prime }} \end{equation*}

where *F_s_* is the steady-state fluorescence level and *F_m_*′ is the fluorescence level during the flash. The NPQ was calculated according to Bilger and Björkman (1990):


(2) \begin{equation*} NPQ=\frac{F_m}{F_m^{\prime }}-1 \end{equation*}

where *F_m_* is the fluorescence level of dark-adapted leaves during the flash, obtained before the induction phase.

The PRI is defined as the normalized reflectance index using 531 nm and 570 nm (Gamon et al. 1993, Peñuelas et al. 1995):


(3) \begin{equation*} PRI=\frac{\rho_{531}-{\rho}_{570}}{\rho_{531}+{\rho}_{570}} \end{equation*}

We analyzed temporal changes in parameters after a change in light intensity. We defined the value at the beginning of each phase (induction, deactivation, and recovery) as the initial value, which was generally measured within 4 s after the change of illumination, except for Φ_P_. PRI was not measured in the dark because reflectivity cannot be measured in the dark. To match the analysis of the PRI with that of other parameters, the value after the change of illumination was selected. For Φ_P_, we defined the initial value as the lowest value after the onset of light because it has a high value in the dark and then decreases very quickly. At least 5 min after each parameter reached a steady state, we obtained the “final value” of each parameter. This was calculated as the average value of the last 30 s of the steady state. Based on the initial and final values, we calculated the time in which the value achieved 50% or 90% of the difference between the final and initial values as IT_50_ and IT_90_, respectively.

### Statistical analysis

Through experiments and the above calculations, dynamic and steady-state parameters were obtained for each individual. Because the measured values of the PRI were often noisy, it was difficult to calculate its half-time when the amplitude of the noise was comparable to the magnitude of the change in the index. Therefore, we excluded data with high PRI noise and selected seven parameter sets from 11 individuals of I-214 and six parameter sets from 11 individuals of Peace for statistical analysis. The Shapiro–Wilk test was performed on each parameter set to assess normality. All response time data were log-transformed to ensure homogeneity of variance. For comparisons among different parameters within the same poplar type, we conducted paired *t*-tests followed by Holm’s post-hoc correction (Holm 1979).

In I-214 leaves, oscillations in stomatal conductance and *A* were observed during induction (Fig. 2a). To evaluate whether PSII parameters were synchronized with these oscillations, we extracted data points for each parameter within the time windows in which *A* decreased and calculated correlation coefficients (*r*) from linear regressions of each parameter against time and *g_s_* (Fig. 4).

To determine whether relationships between variables differed among phases, we employed an LMM using the “nlme” package in R. In the model, one variable was treated as the dependent variable, the other was treated as a continuous fixed effect, phase (induction, recovery, steady-state) was included as a categorical fixed effect, and individual leaf identity was treated as a random effect.

## Supplementary Material

pcp-2025-e-00068-File011_pcaf111

## Data Availability

The datasets generated in the present study are available from the corresponding author on reasonable request.
