# Supplementary material for: Photochemical reflectance index and its relation to photosynthetic characteristics under dynamic light environment
Source: Plant Cell Physiol. 2025 Sep 11;66(11):1760–71. doi: 10.1093/pcp/pcaf111 (PMC12661318; doi:10.1093/pcp/pcaf111)
Supplement: pcp-2025-e-00068-File011_pcaf111 [file pcp-2025-e-00068-file011_pcaf111.pdf]

Table S1 Relationship between Ocean HR exposure time and PPFD of the illumination LED during measurement.

PPFD (μmol m^{-2} s^{-1})	Exposure time (ms)
100	3000
200	1600
300	1050
400	800
500	600
1000	300
1500	230

Figure S1

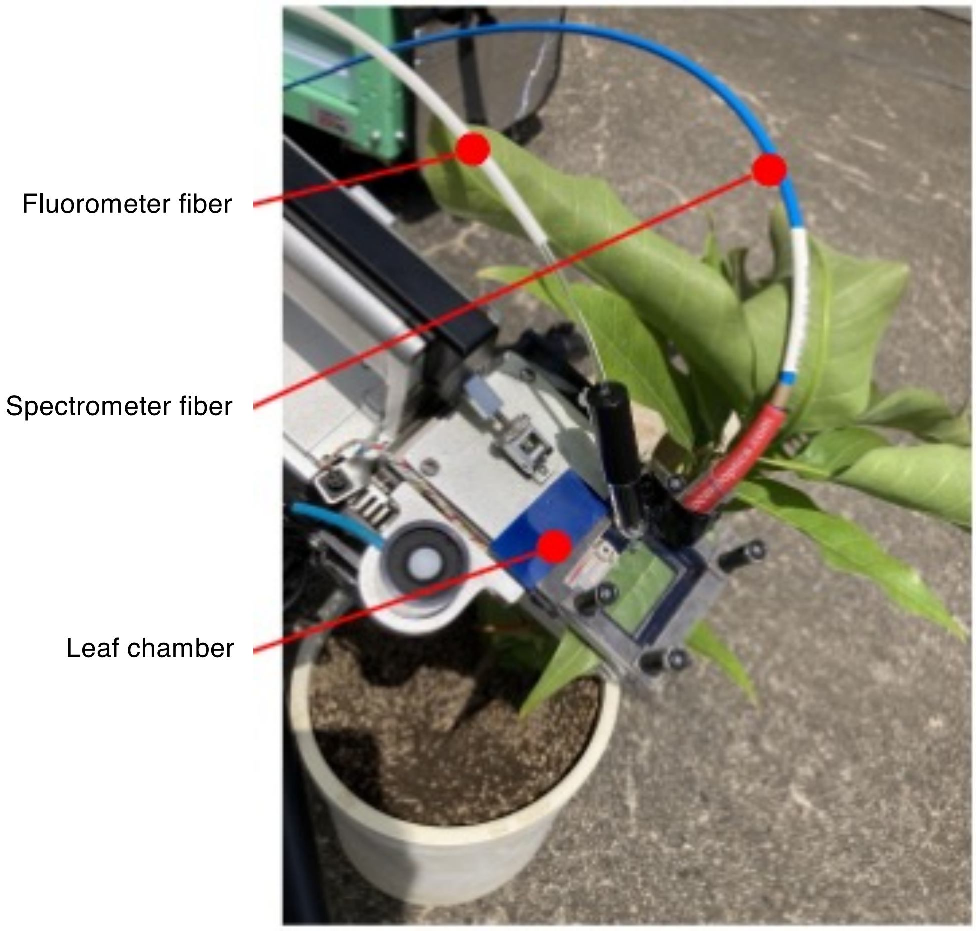

Fig. S1 Measurement system setup. Li-6400 is used to measure gas exchange. The fluorometer fiber is connected to MINI-PAM-II to measure the state of the photosystem. The spectrometer fiber is connected to the High Resolution Spectrometer to measure the reflectance spectrum.

Figure S2

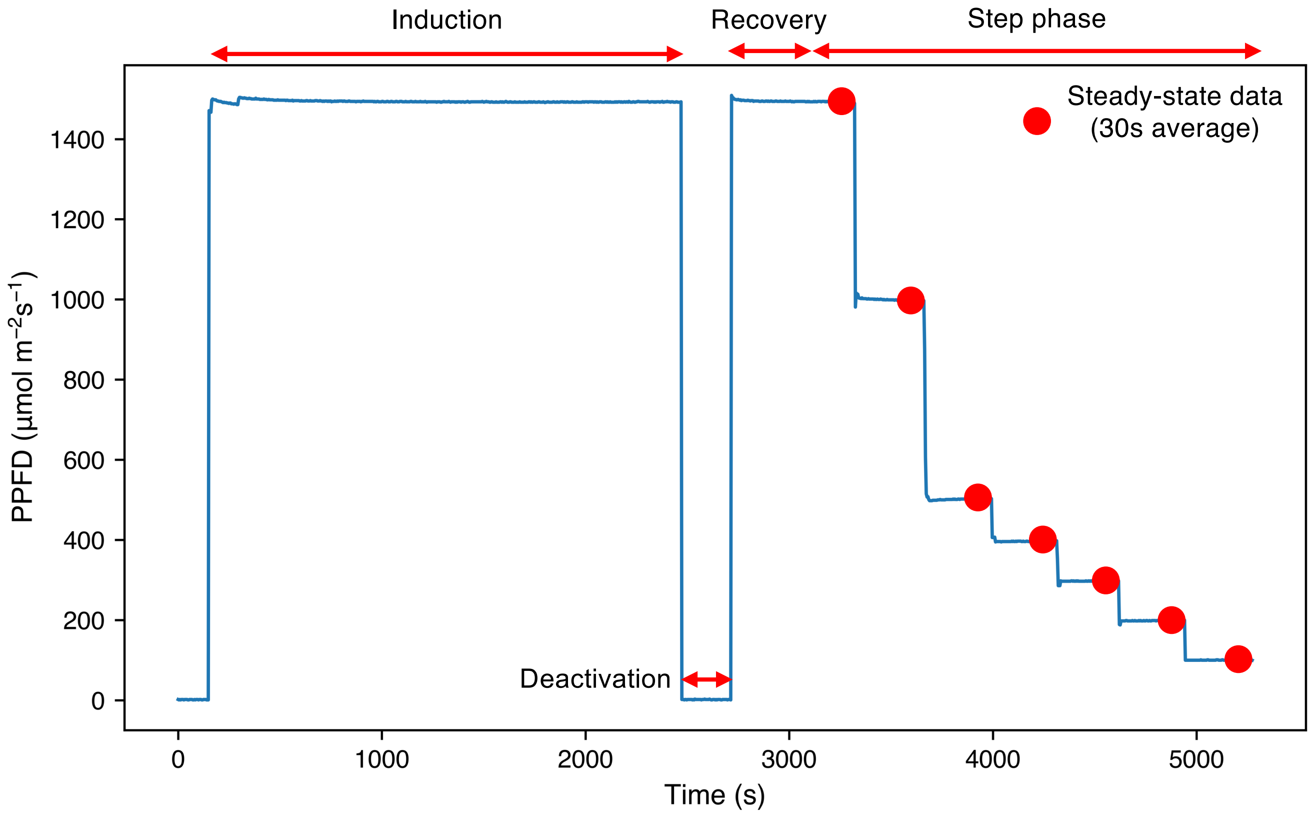

Fig. S2 Example of illumination. Initial 2 minutes of darkness, followed by induction phase, deactivation phase, recovery phase, and step phase. The duration of the induction phase and recovery phase varies for different individuals to ensure that photosynthesis reaches a steady state for each individual. The deactivation phase is 4 minutes. The step phase is set with illumination gradients, each lasting 5 minutes.

Figure S3

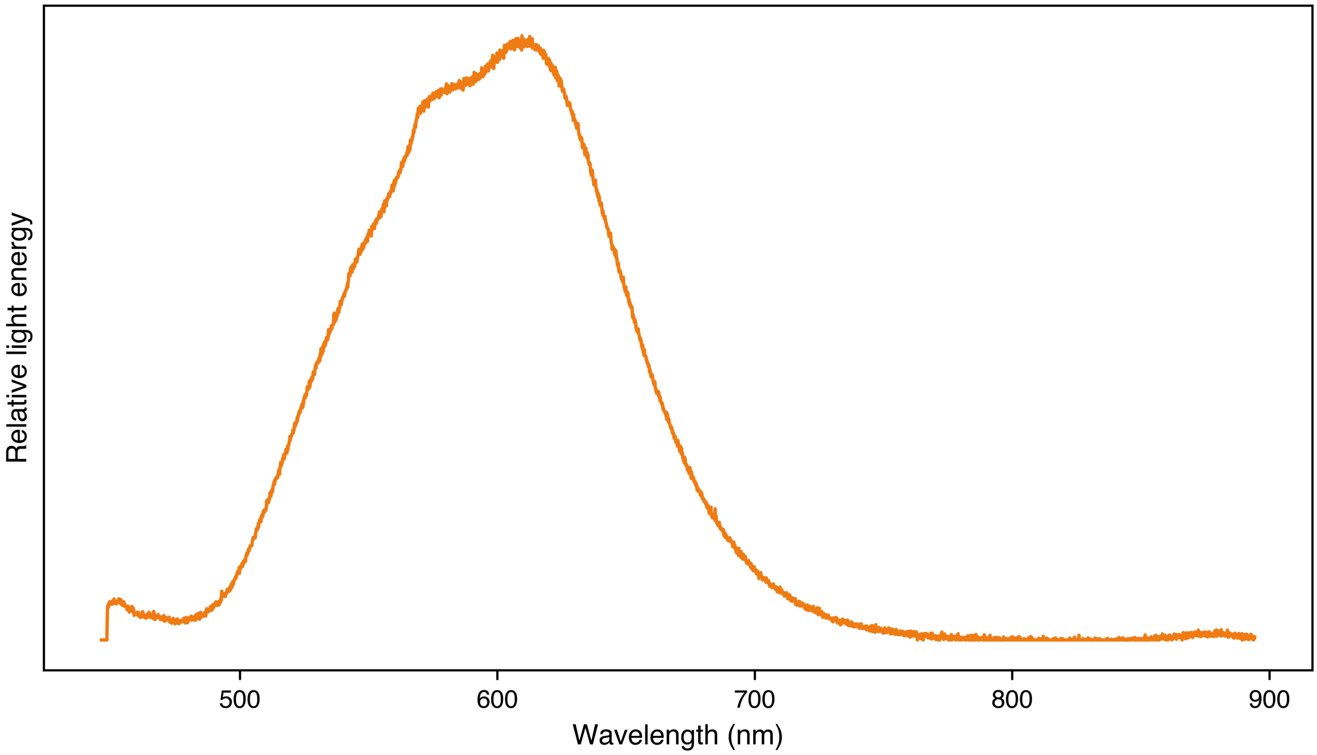

Fig. S3 Illumination spectrum.
